# Energy expenditure and dietary intake in research: A visualization analysis

**DOI:** 10.1177/02601060251404993

**Published:** 2026-01-19

**Authors:** Sarah A. Craven, Grace A. Suter, Erin D. Giles, Sarah A. Purcell

**Affiliations:** 1 School of Health and Exercise Sciences, 97950 University of British Columbia Okanagan, Kelowna, Canada; 2 School of Kinesiology, 1259 University of Michigan, Ann Arbor, USA; 3 Department of Medicine, University of British Columbia, Vancouver, Canada; 4 Centre for Chronic Disease Prevention and Management, University of British Columbia Okanagan, Kelowna, Canada

**Keywords:** Metabolism, bibliometrics, nutrition, food intake, animal models, dietary intake, obesity, human models

## Abstract

**Background:**

Assessing energy expenditure (EE) is critical for identifying dietary intake (DI) requirements for nutrition care and management. Understanding both EE and DI can provide deeper insight into energy balance, which is crucial considering the rising prevalence of obesity.

**Objective:**

The objective of this visualization and bibliometric analysis was to characterize emerging trends in EE and DI literature from 2013 to 2023.

**Methods:**

Web of Science Core Collection was searched using EE- and DI-related terms. A manual screening process was used to enhance the relevancy of included articles and to dichotomize articles as animal or human research.

**Results:**

7149 articles were included: 4130 focused on animal models and 3462 focused on humans. Annual new publications grew 8.1% from 2013 to 2022. New publications increased faster in animal research at 10.1% compared to only 4.7% in human research. Keywords in animal and human domains clustered around four topics: ‘energy expenditure’, ‘metabolism mechanisms’, ‘obesity’, and ‘dietary intake’. The most frequent keyword was ‘obesity’ in both animal and human research.

**Conclusions:**

Animal and human research saw different trends in the rate of annual new publications, highly cited references, and keywords, highlighting the distinct approaches in animal and human models within EE and DI research.

## Introduction

The global obesity epidemic represents one of the most pressing public health challenges of the twenty-first century (World Health Organization, 2025), driving increased rates of cardiovascular disease, diabetes, and related chronic health conditions worldwide. Addressing this complex epidemic requires an in-depth understanding of the underlying drivers of energy balance, specifically dietary intake (DI) and energy expenditure (EE). Understanding the interplay between these two factors is critical for developing effective intervention strategies to prevent and manage obesity and its associated comorbidities.

EE reflects the body's total energy utilization, including the energy required to support resting metabolism, physical activity, and diet-induced thermogenesis (Fernández-Verdejo et al., 2024). In contrast, DI represents the total energy, macronutrients, and micronutrients consumed. Despite broad recognition of the importance of energy balance, DI guidelines for chronic disease management and optimal health vary widely across countries, with no global consensus (Koliaki et al., 2018). Accurate measurement and prediction of EE are essential for assessing energy requirements and optimizing nutrition care in the management of obesity and other chronic conditions (Committee on the Dietary Reference Intakes for Energy, Food and Nutrition Board, Health and Medicine Division 2023; Wharton et al., 2020), and can inform the evaluation of intervention effectiveness (Lim et al., 2024). Characterizing both EE and DI allows for a more nuanced understanding of energy balance, which is especially important in the context of the current global obesity crisis.

Obesity is now the most prevalent chronic condition in high-income nations and is rapidly rising in low- and middle-income countries as well (Murray et al., 2020; Okunogbe et al., 2022; World Health Organization, 2025). Thus, a comprehensive understanding of the recent scientific literature related to EE and DI is needed to uncover publication deficits, knowledge gaps, and research funding priorities to support global efforts in mitigating obesity and related conditions.

Over the past decade, researchers have employed a variety of literature synthesis methods to explore aspects of EE and DI (Foo et al., 2025; González-Gross et al., 2021; Mehranfar et al., 2024; Purcell et al., 2021; Reed et al., 2024). Complementary to systematic, narrative, and scoping reviews, bibliometric analyses provide an established method for analyzing and visualizing research landscapes (Donthu et al., 2021). Previous bibliometric reviews have examined DI in relation to specific health outcomes (Zyoud, Shakhshir et al., 2022), disease states (Giles et al., 2023), and diet approaches (Liu and Lu, 2023; Lu et al., 2023; Wang et al., 2022); however, none to date have assessed DI in relation to EE. A bibliometric analysis at the intersection of EE and DI is particularly timely given the rapid evolution of this globally relevant field.

Both preclinical (animal) and human studies offer unique insight into understanding EE. Animal studies often elucidate mechanistic processes not easily examined in humans. On the other hand, human research primarily involves clinical trials and implementation efforts. Examining bibliometric patterns across both animal and human studies can help identify research trends and emerging areas of interest.

The aim of this review is to identify emerging trends and knowledge gaps in EE and DI research using bibliometric methods. Specifically, we analyze publication growth, country and journal distribution, highly cited references, and keyword trends from 2013 to 2023, comparing developments in both animal and human research models to provide a novel overview of the current field.

## Methods

### Study design

Bibliometric and visualization analyses provide both qualitative and quantitative overviews of the scope of publications within a research field by leveraging publication metadata and citation metrics (Agarwal et al., 2016; Donthu et al., 2021). Unlike systematic reviews and meta-analyses, which critically appraise and summarize data on a specific topic, bibliometric approaches summarize publication trends, performance metrics, and map co-occurrences to highlight prominent areas and knowledge gaps within the literature (Donthu et al., 2021). In this study, we followed published bibliometric methodology and reporting guidelines (Romanelli et al., 2021). Ethical approval was not required as the study exclusively used publicly available information and published materials.

### Search strategy

The Web of Science Core Collection (WoSCC; Clarivate Analytics, USA) was used to identify relevant publications. WoSCC is among the most influential databases for health-related bibliometric analyses (Merigó and Núñez, 2016; Pranckutė, 2021) due to its broad disciplinary coverage and its accuracy in document classification and citation retrieval (Falagas et al., 2008; Kulkarni et al., 2009; Romanelli et al., 2021; Yeung, 2019).

The search strategy for EE and DI-related terms was developed by the research team through an iterative process informed by domain expertise and preliminary searches, with refinements based on the relevance of the first 10–50 returned articles. Additional diet and nutrition terms (e.g., nutrient* OR feed* OR diet NEAR/5 intake OR nutrition NEAR/5 intake OR “nutritional status” OR undernutrition* OR overfeed*) were piloted but ultimately excluded as search results contained many publications that were not relevant. Search terms were intentionally broad to generate a comprehensive qualitative and quantitative overview of the research field (Donthu et al., 2021). The search and data extraction were conducted on August 1, 2023. The final WoSCC query included EE and DI-related terms in the title and abstract as follows:

(((AB = (energy NEAR/5 metabolism OR energy NEAR/5 expenditure OR “metabolic rate” OR “caloric expenditure” OR “calorie expenditure” OR thermogen*)) OR (TI = (energy NEAR/5 metabolism OR energy NEAR/5 expenditure OR “metabolic rate” OR “caloric expenditure” OR “calorie expenditure” OR thermogen*))) AND (AB = (nutrition* OR diet*) OR TI = (nutrition* OR diet*))) NOT (TI = (review) OR (AB = (review)).

The search was limited to articles published between 2013 and 2023 to focus on recent and relevant publication trends, as previously recommended (Abdelwahab et al., 2025; Romanelli et al., 2021).

### Screening of search results

Although not required for visualization analyses, manual screening of publications is recommended to enhance the rigor and relevance of bibliometric analyses (Romanelli et al., 2021). Search results were uploaded to Covidence (Vertitas Health Innovation, Australia) for eligibility screening. Eligible studies were English-language publications clearly related to EE and DI in animal or human research. Studies on brown adipose tissue and metabolism of isolated substrates (i.e., lipid, glucose, protein, etc.) were included if additional DI factors were discussed. Publications describing animal studies of EE, nutrition, and/or DI for purposes not relevant to humans (i.e., agriculture, livestock, veterinary medicine) were excluded to ensure analytical consistency. Proceeding papers, meeting abstracts, editorials, and book chapters were also excluded.

The screening protocol was developed and piloted in line with previous bibliometric reviews (Huang et al., 2022; Müller et al., 2018), and two co-authors (SAC and GAS) and two research assistants independently screened publication titles for eligibility. If eligibility could not be determined by title alone, abstracts and/or full-text were reviewed. The process also included assigning studies as animal or human research. Studies involving animals for clinical or biomedical purposes related to EE and DI were classified as animal research (e.g., interventions, longitudinal data, case studies, mathematical modeling, *ex-vivo* tissue studies, etc.), while all others were designated as human research.

To ensure consistency among screeners, each screener evaluated a training set of 30 papers until a Cronbach's alpha of at least 0.81 was reached. Screening continued independently until all remaining papers were evaluated by at least two screeners. Regular meetings were held to discuss screening consistency and resolve disagreements; unresolved cases were reviewed by an additional co-author (SAP or EDG).

### Data analysis and visualization

Data analysis and visualization were conducted using Microsoft Excel (Version 2507), the bibliometrix package for RStudio (Version 4.2.3), and VOSViewer software (version 1.6.20). Full records and cited references were downloaded from WoSCC in plain text format. All analyses were performed separately on dichotomized datasets (animal vs human research) as well as on the combined datasets.

The annual publication growth rate from 2013–2022 was calculated in Excel as:

$$\begin{array}{r} (\mathrm{number}\;\mathrm{of}\;\mathrm{publications}\;\mathrm{in}\;\mathrm{given}\;\mathrm{year}\;\text{-}\\ \frac{\;\;\mathrm{number}\;\mathrm{of}\;\mathrm{publications}\;\mathrm{in}\;\mathrm{previous}\;\mathrm{year})}{\mathrm{number}\;\mathrm{of}\;\mathrm{publications}\;\mathrm{in}\;\mathrm{previous}\;\mathrm{year}} \end{array}$$

The bibliometrix package was used to analyze publication and citation metrics, including trends in top contributing journals, countries, and highly cited references.

VOSViewer was used for visual network analyses of country collaboration and keyword-identified research themes using the full counting method, where all co-authorships and co-occurrences are equally weighted. Both author-derived and WoSCC-derived keywords were included in the keyword co-occurrence analysis. For network visualizations, thresholds were set to include approximately 50 countries and 100 keywords per figure for readability. In these visualizations, node size represents the number of publications for each country or keyword, line thickness represents the strength of collaboration or co-occurrence, and distance between nodes reflects relatedness. Clusters of related keywords were identified using modularity-based clustering within VOSviewer and were manually renamed according to the most common and relevant keywords.

## Results

### Average annual publication trends

A total of 9797 publications were identified in WoSCC at the time of search. Nine duplicate publications were automatically removed during import to Covidence, leaving 9788 publications to be screened. During the screening process, five additional duplicates were identified and manually removed. After manual screening, 7592 publications related to EE and some aspect of DI in animal or human models were retained. To focus on primary research, publications tagged as book chapters, data papers, corrections, editorial materials, letters, meeting abstracts, retractions, reviews, and proceedings papers were excluded. Ultimately, 7149 research articles were included in the final analysis: 4130 used animal models, while 3462 reported data from studies in humans. A flow diagram of the article selection process is provided in supplementary file 1.

**Figure 1.**
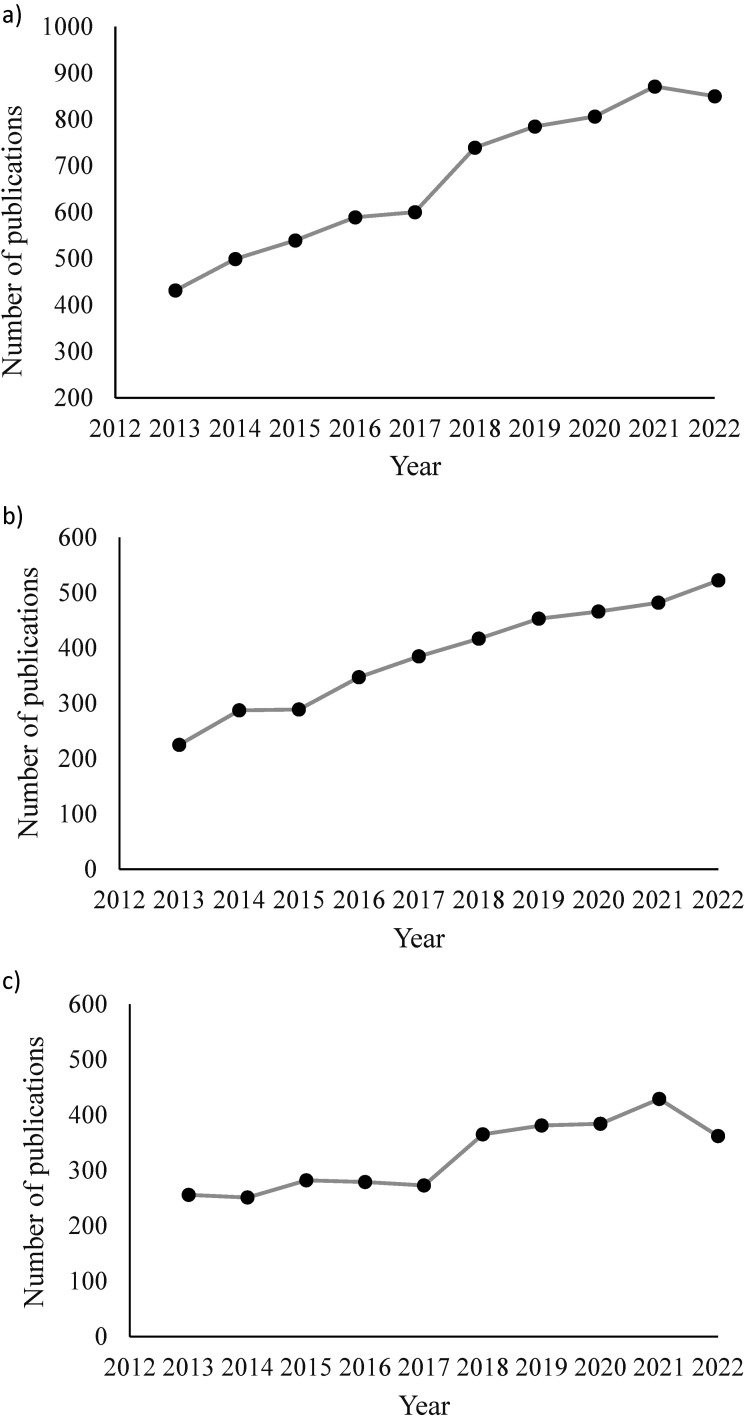
Annual publication growth rate: a) all publications, b) animal research, c) human research.

The number of new publications steadily increased from 2013 to 2022, with an average annual publication growth rate of 8.1% (Figure 1(a)). In 2013 there were more publications on human research (n = 256) than animal research (n = 225); however, the higher growth rate for animal research compared to human research over the time (10.1% vs 4.7%, respectively) resulted in more animal-related publications by 2022 (n = 522) than human studies (n = 362; Figures 1(b) and 1(c)).

### Distribution by country

Articles related to EE and DI were published by authors from 110 different countries; all of these countries were represented in publications of human data, while animal research only included authors from 79 countries; see supplementary file 2a. The United States contributed the largest number of animal model publications, followed by China, and Japan. For human research, the top three countries were the United States, followed by China, and Brazil.

A co-authorship analysis map (supplementary file 2b) was constructed to illustrate international collaboration and publication output. The United States, England, China, Germany, and Canada demonstrated the strongest international research collaborations within this field.

### Distribution by journal

Research related to EE and DI was published in 1360 different journals between 2013 and 2023. Animal model research appeared in 711 journals, while human research was published in 1098 journals. Among animal model studies, the top journals by publication count were *PLOS One*, *Molecular Metabolism*, and *Nutrients* (Table 1). For human research, *Nutrients* published the most papers, followed by *Clinical Nutrition* and *PLOS One*. For animal studies, *Diabetes* was the most highly cited journal, followed by *PLOS One* and *Molecular Metabolism*. In human research, the *American Journal of Clinical Nutrition* had the highest citation count, followed by *Nutrients* and *Clinical Nutrition*. Impact factors (IF) for the top journals ranged from 3.7–8.1 for animal research and from 3.6–7.1 for human research.

**Table 1.** Top 10 journals with the most publications.

**Journals related to animal research**			
Journal	Publications (%)	Citation	IF (2022)
PLOS One	154 (3.7)	4728	3.7
Molecular Metabolism	141 (3.4)	3953	8.1
Nutrients	123 (3.0)	1964	5.9
Scientific Reports	114 (2.8)	2671	4.6
Diabetes	110 (2.7)	4786	7.7
Journal of Nutritional Biochemistry	107 (2.6)	2666	5.6
American Journal of Physiology-Endocrinology and Metabolism	97 (2.3)	2277	5.1
International Journal of Molecular Sciences	79 (1.9)	835	5.6
International Journal of Obesity	76 (1.8)	2575	4.9
FASEB Journal	74 (1.8)	1401	4.8

**Journals related to human research**			
Journal	Publications (%)	Citation	IF (2022)
Nutrients	199 (5.7)	2255	5.9
Clinical Nutrition	96 (2.8)	2311	6.3
PLOS One	79 (2.3)	1907	3.7
American Journal of Clinical Nutrition	71 (2.1)	2576	7.1
Clinical Nutrition (ESPEN)	58 (1.7)	1351	6.3
Journal of Nutrition	54 (1.6)	254	4.2
British Journal of Nutrition	53 (1.5)	1086	3.6
Nutrition	48 (1.4)	584	5.4
Obesity	45 (1.3)	1173	6.9
International Journal of Obesity	42 (1.2)	1508	4.9

### References within manuscripts

The 7149 papers included in our analysis cited a total of 207,511 references: 120,854 in animal model papers and 121,628 in human papers. The top 10 cited references in each category are presented in Table 2**.** Cannon & Nedergaard (Cannon and Nedergaard, 2004) was the most frequently cited by animal papers, followed by Cypess et al. (Cypess et al., 2009) and Wu et al. (Wu et al., 2012). In human research the most cited references were Weir (Weir, 1949), followed by Harris & Benedict (Harris and Benedict, 1918) and Mifflin (Mifflin et al., 1990). Overall, the papers most often cited by animal studies were those focused on brown adipose tissue, while papers highly cited by clinical research were primarily focused on the measurement of EE in humans.

**Table 2.** Top 10 most cited references.

**References from animal research**				
Rank	Title	Author	Year	Citations
1	Brown adipose tissue: function and physiological significance (Cannon and Nedergaard, 2004)	Cannon & Nedergaard	2004	414
2	Identification and importance of brown adipose tissue in adult humans (Cypess et al., 2009)	Cypess et al.	2009	289
3	Beige adipocytes are a distinct type of thermogenic fat cell in mouse and human (Wu et al., 2012)	Wu et al.	2012	289
4	Brown and beige fat: development, function and therapeutic potential (Harms and Seale, 2013)	Harms & Seale	2013	283
5	Cold-activated brown adipose tissue in healthy men (van Marken Lichtenbelt et al., 2009)	van Marken Lichtenbelt et al.	2009	222
6	A simple method for the isolation and purification of total lipides from animal tissues (Folch et al., 1957)	Folch et al.	1957	221
7	UCP1 ablation induces obesity and abolishes diet-induced thermogenesis in mice exempt from thermal stress by living at thermoneutrality (Feldmann et al., 2009)	Feldmann et al.	2009	192
8	Functional brown adipose tissue in healthy adults (Virtanen et al., 2009)	Virtanen et al.	2009	191
9	Analysis of relative gene expression data using real-time quantitative PCR and the 2(-DeltaDelta C(T)) Method (Livak and Schmittgen, 2001)	Livak & Schmittgen	2001	181
10	Prdm16 determines the thermogenic program of subcutaneous white adipose tissue in mice (Seale et al., 2011)	Seale et al.	2011	163

**References from human research**				
Rank	Title	Author	Year	Citations
1	New methods for calculating metabolic rate with special reference to protein metabolism (Weir, 1949)	Weir	1949	395
2	A biometric study of human basal metabolism (Harris and Benedict, 1918)	Harris & Benedict	1918	182
3	A new predictive equation for resting energy expenditure in healthy individuals (Mifflin et al., 1990)	Mifflin et al.	1990	176
4	Predicting basal metabolic rate, new standards and review of previous work (Schofield, 1985)	Schofield	1985	145
5	Best practices methods to apply to measurement of resting metabolic rate in adults: a systematic review (Compher et al., 2006)	Compher et al.	2006	131
6	Homeostasis model assessment: insulin resistance and beta-cell function from fasting plasma glucose and insulin concentrations in man (Matthews et al., 1985)	Matthews et al.	1985	114
7	Calculation of substrate oxidation rates in vivo from gaseous exchange (Frayn, 1983)	Frayn	1983	111
8	Statistical methods for assessing agreement between two methods of clinical measurement (Bland and Altman, 1986)	Bland & Altman	1986	96
9	Changes in energy expenditure resulting from altered body weight (Leibel et al., 1995)	Leibel & Hirsch	1995	81
10	Comparison of predictive equations for resting metabolic rate in healthy nonobese and obese adults: a systematic review (Frankenfield et al., 2005)	Frankenfield et al.	2005	75

### Keywords

**Figure 2.**
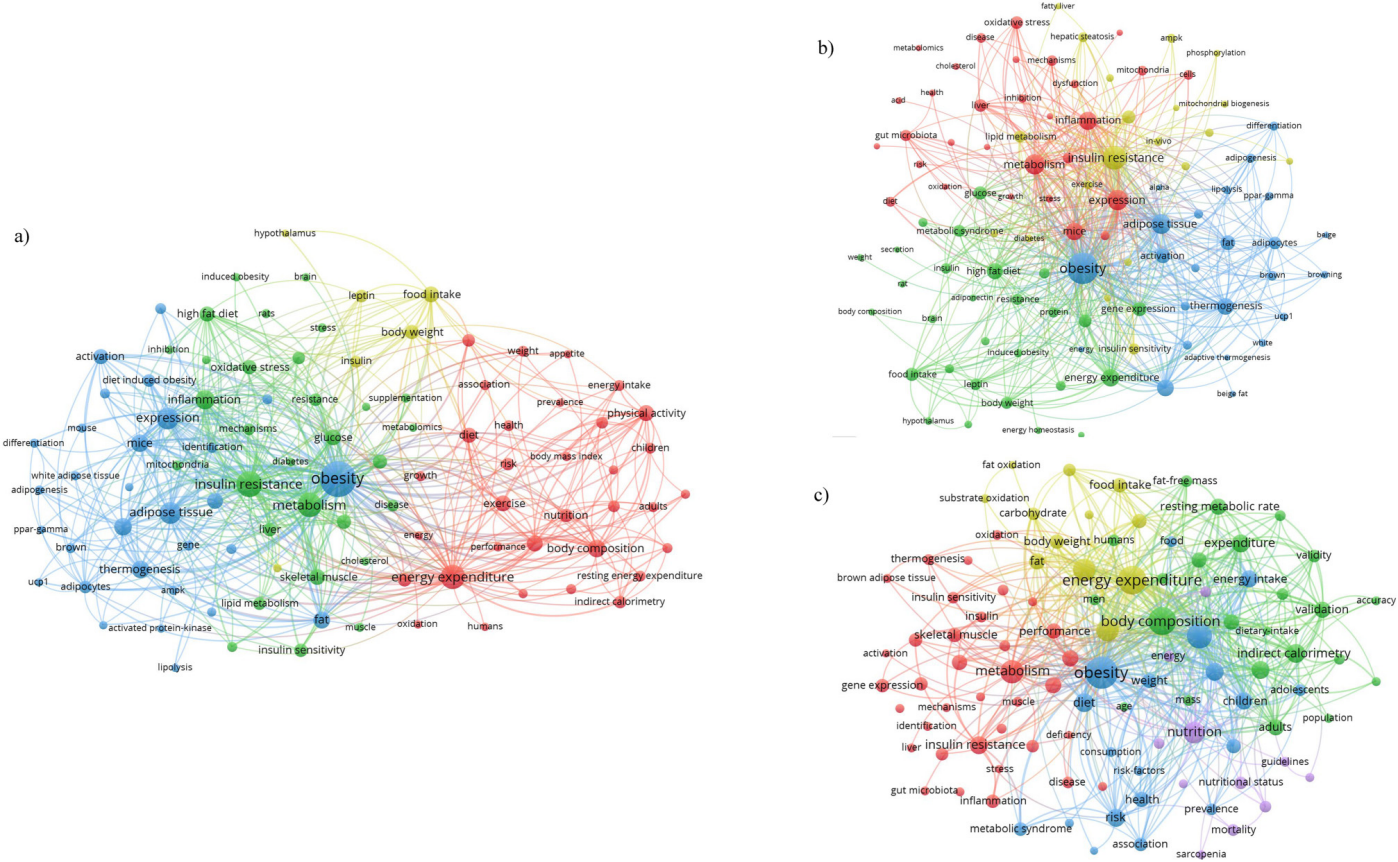
Co-occurrence analysis of the top 100 keywords: a) all publications, b) animal research, c) human research.

There were 20,141 unique keywords across all included publications. Figure 2(a) illustrates the top 100 keywords, which clustered into four thematic groups: energy expenditure (red), metabolism mechanisms (green), obesity (blue), and dietary intake (yellow). Highly prevalent keywords included: “obesity” (2201 occurrences), “energy expenditure” (1005 occurrences), “metabolism” (997 occurrences), “thermogenesis” (534 occurrences), and “food intake” (431 occurrences).

Figures 2(b) and 2(c) illustrate the top 100 keywords in animal and human research, respectively. In these subgroup analyses, keywords formed denser, more circular clusters compared to Figure 2(a), indicating closer thematic relationships when separated by research model. Animal research keywords (n = 11,797) formed four clusters: metabolism and inflammation (red), high fat diet (green), obesity (blue), and insulin resistance (yellow). Human research keywords (n = 11,412) formed five clusters: metabolism/metabolic markers (red), energy expenditure methodology and body composition (green), obesity (blue), energy expenditure and weight loss (yellow), and nutrition (purple).

“Obesity” was the most frequently assigned keyword in both animal (1561 occurrences) and human (640 occurrences) research. In animal model publications, other frequently used keywords included “insulin resistance” (923 occurrences), “adipose tissue” (630 occurrences), “mice” (500 occurrences), and “high fat diet” (406 occurrences). For human research, commonly used keywords were “body composition” (457 occurrences), “physical activity” (392 occurrences), “weight loss” (319 occurrences), “nutrition” (285 occurrences), and “insulin resistance” (202 occurrences). Complete tables of the top 100 keywords can be found in supplementary file 3.

## Discussion

This bibliometric and visualization analysis provides an overview of evolving trends in EE and DI research. We observed steady publication growth over the past decade, primarily driven by a rapid increase in animal studies, potentially due to the relative feasibility and shorter timelines of these studies compared to those involving humans. ‘Obesity’ was among the most common keywords in both animal and human studies, underscoring the central and critical role of EE and DI in obesity research. Notably, the decline in publication rates of human studies from 2021–2022 may reflect disruptions caused by the COVID-19 pandemic, which negatively impacted study visits involving humans, participant recruitment, supply chain, and allocation of funding (Sohrabi et al., 2021).

While animal studies remain invaluable for uncovering mechanistic insights that cannot be easily investigated in humans, some aspects of EE research involving complex behavioral, environmental, and social influences may be better addressed in human studies because of their direct relevance and applicability. As the field progresses, it will become increasingly important to balance the strengths of animal research, such as experimental control and mechanistic exploration, with the strengths of human research, including direct translatability and contextual relevance. Integrating findings from both approaches will be critical for advancing our understanding of EE and DI in real-world contexts.

The United States and China led EE and DI research output and demonstrated the strongest international collaboration, findings consistent with other nutrition-related bibliometric studies (Huang et al., 2022; Liu and Lu, 2023; Lu et al., 2023; Müller et al., 2018; Wang et al., 2022; Zyoud, Al-Jabi et al., 2022; Zyoud, Shakhshir et al., 2022). Research output from developing countries remains limited. Promoting collaboration and sharing resources with emerging contributors such as New Zealand, India, Switzerland, Italy, and Mexico, may broaden perspectives and innovation in the field.

Patterns in journal publication highlight the interdisciplinary nature of EE and DI research. Animal studies were most often published in journals with a focus on molecular biology and biochemistry, while human studies appeared more frequently in nutrition-focused journals. This distribution reflects the differing research priorities and audiences associated with each domain. Notably, no single journal emerged as the preeminent publisher of animal EE and DI research, which may reflect the broad interdisciplinarity of the field and tendency for authors to select journals tailored to specific readerships. In contrast, *Nutrients* accounted for the largest proportion of human EE and DI publications. This finding aligns with other bibliometric analyses in the diet and nutrition space, which similarly identified *Nutrients* as a top publisher in this area (Huang et al., 2022; Liu and Lu, 2023; Lu et al., 2023; Wang et al., 2022; Zyoud, Al-Jabi et al., 2022; Zyoud, Shakhshir et al., 2022). *Diabetes* and *American Journal of Clinical Nutrition* published marginally fewer articles but had the highest impact factors for both animal and human research, respectively. This lower volume may reflect the competitiveness and selectivity of high impact factor journals.

Highly cited references are widely recognized as foundational contributions within a research field. In our analysis, there was a clear distinction between the most cited references in animal and human research. Among the top 10 most highly cited articles, those referenced by animal studies included many papers related to brown adipose tissue, whereas the highly cited articles in human research centered on the measurement of EE. References cited in animal research tended to be more recent, with most published between 2001 and 2013. This trend likely reflects the relatively recent emergence of brown adipose tissue as a topic of interest and area of advancing innovation. In contrast, the most frequently cited references in human research were considerably older, predominantly from 1949 to 1995. The continued citation of these older works highlights the widespread acceptance of fundamental techniques that continue to be integral to the field. For example, the most cited reference for human research was the first publication of the Weir equation (Weir, 1949), which calculates EE using measurements of oxygen consumption and carbon dioxide production. This equation remains a cornerstone in EE research.

Keywords often serve to identify core topics and indicate major areas of interest. ‘Obesity’ emerged as a central keyword in both animal and human studies, which aligns with the high prevalence and global burden of obesity (Abarca-Gómez et al., 2017), as well as its prominence as a research priority among health authorities, including the World Health Organization. The inherently interdisciplinary nature of obesity research is evident in our findings. For example, animal studies commonly linked ‘obesity’ to molecular mechanisms such as ‘adipose tissue’, ‘thermogenesis’, and ‘brown adipose tissue’, whereas human studies more often associated ‘obesity’ with factors such as ‘physical activity’, ‘diet’, and ‘energy intake’. These patterns likely reflect the different emphases of EE and DI research in animal studies, which focus on mechanistic understanding, and in human studies, which prioritize population health and well-being.

Identifying areas of overlap and divergence in keyword usage can guide cross-disciplinary integration and help bridge animal and human research to advance EE and DI understanding. While central keywords reflect current research focuses, peripheral keywords may signal subfields and emerging areas of focus. Keywords at the periphery of animal research included ‘substrate oxidation’, ‘adipose tissue browning’, and ‘gut microbiota’. In human research, peripheral keywords included ‘validation’, ‘metabolic syndrome’, and ‘brown adipose tissue’. The identification of central and peripheral keywords supports researchers and policy makers in identifying future research directions and funding priorities.

There are several strengths and limitations to consider in interpreting our results. Unlike previous analyses that relied on an automated screening, we manually reviewed the titles and abstracts of the 9788 publications identified in the initial search to improve the specificity and relevance of included articles. We also conducted separate analyses for animal and human research, allowing for a more nuanced understanding of trends in each sub-field. However, limiting our search to the WoSCC database means some relevant articles from other databases may not have been captured, a known limitation of bibliometric studies (Falagas et al., 2008). Manual categorization of papers into animal and human research also carries the potential for misclassification. Lastly, while not a limitation of our study design, it remains a challenge in the field that research output is dominated by a small number of large countries; future research should therefore prioritize the global contributions to research in this field.

## Conclusions

By applying best practices for bibliometric analyses, we identified emerging trends in EE and DI research, including publication characteristics, international collaborations, journal distribution, frequently cited references, and keyword patterns. Separating animal and human studies revealed distinct differences in annual publication rates, highly cited references, and key research topics, highlighting the distinct approaches in each domain. Obesity was the most prominent keyword overall, underscoring its central importance and potential for future cross-disciplinary collaboration. Future research should focus on expanding collaborations with countries where EE and DI research is emerging, exploring areas for cross-disciplinary research in animal and human research, and monitoring peripheral subfields to identify new directions for the field.

## Supplemental Material

sj-pdf-1-nah-10.1177_02601060251404993 - Supplemental material for Energy expenditure and dietary intake in research: A visualization analysis Supplemental material, sj-pdf-1-nah-10.1177_02601060251404993 for Energy expenditure and dietary intake in research: A visualization analysis by Sarah A. Craven, Grace A. Suter, Erin D. Giles and Sarah A. Purcell in Nutrition and Health

sj-pdf-2-nah-10.1177_02601060251404993 - Supplemental material for Energy expenditure and dietary intake in research: A visualization analysis Supplemental material, sj-pdf-2-nah-10.1177_02601060251404993 for Energy expenditure and dietary intake in research: A visualization analysis by Sarah A. Craven, Grace A. Suter, Erin D. Giles and Sarah A. Purcell in Nutrition and Health

sj-pdf-3-nah-10.1177_02601060251404993 - Supplemental material for Energy expenditure and dietary intake in research: A visualization analysis Supplemental material, sj-pdf-3-nah-10.1177_02601060251404993 for Energy expenditure and dietary intake in research: A visualization analysis by Sarah A. Craven, Grace A. Suter, Erin D. Giles and Sarah A. Purcell in Nutrition and Health

## List of abbreviations

EE: energy expenditure
DI: dietary intake
WoSCC: Web of Science Core Collection
