# Supplementary figures and images for: Energy expenditure and dietary intake in research: A visualization analysis
Source: Nutr Health. 2026 Jan 19;32(3):777–86. doi: 10.1177/02601060251404993 (PMC13144660; doi:10.1177/02601060251404993)

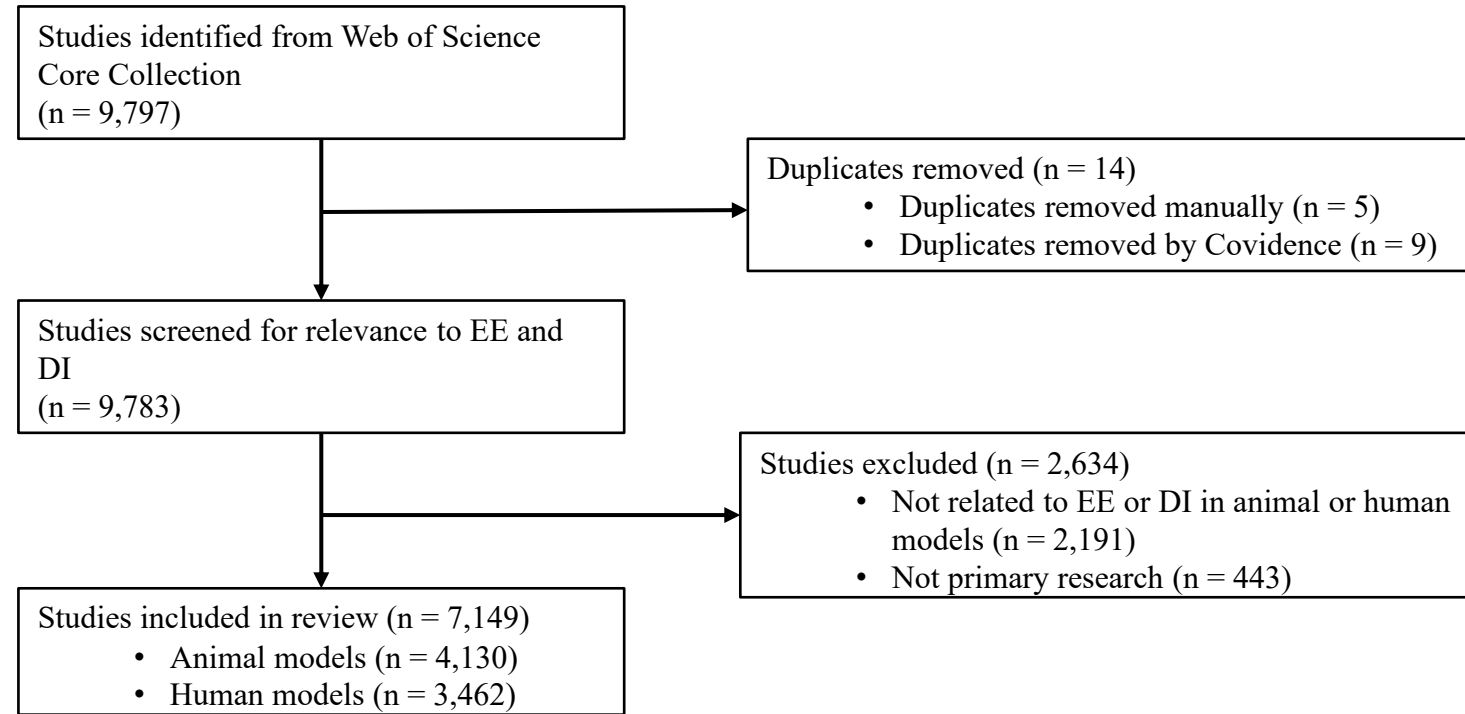

**Supplemental file 1.** Flow diagram of article selection. EE, energy expenditure; DI, dietary intake.

Supplement: sj-pdf-1-nah-10.1177_02601060251404993 - Supplemental material for Energy expenditure and dietary intake in research: A visualization analysis [file sj-pdf-1-nah-10.1177_02601060251404993.pdf]
