# Supplementary material for: Energy expenditure and dietary intake in research: A visualization analysis
Source: Nutr Health. 2026 Jan 19;32(3):777–86. doi: 10.1177/02601060251404993 (PMC13144660; doi:10.1177/02601060251404993)

Supplemental file 2a. Top 10 productive countries

Animal research

Rank	Country	Publications (N)	Percentage (N/4130)
1	United States	1138	27.7
2	China	933	22.7
3	Japan	278	6.7
4	Korea	277	6.7
5	Brazil	177	4.3
6	Canada	146	3.6
7	Germany	144	3.5
8	Spain	144	3.5
9	France	116	2.8
10	Australia	86	2.1

Human research

Rank	Country	Publications (N)	Percentage (N/3462)
1	United States	751	22
2	China	346	10.2
3	Brazil	211	6.2
4	United Kingdom	204	6
5	Italy	164	4.8
6	Canada	159	4.7
7	Japan	155	4.6
8	Spain	149	4.4
9	France	124	3.6
10	Australia	119	3.5

Supplemental file 2b. Co-authorship analysis of the countries with the greatest number of publications.

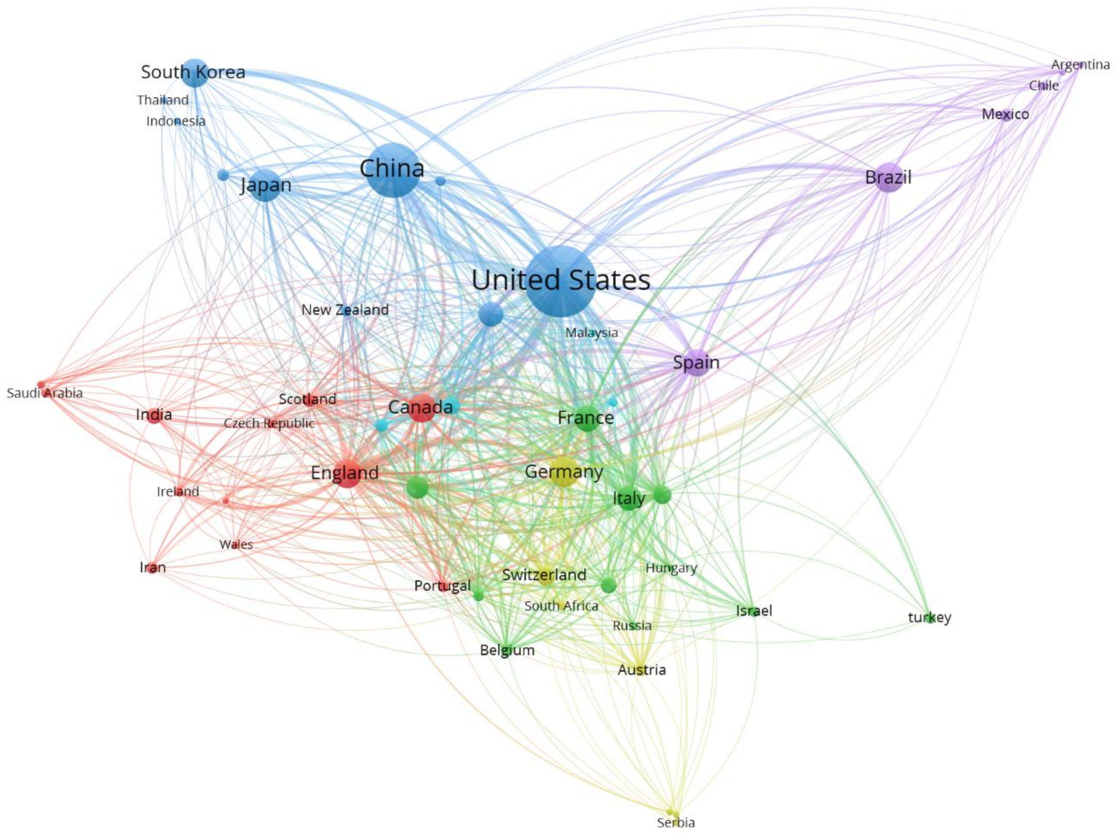

Supplement: sj-pdf-2-nah-10.1177_02601060251404993 - Supplemental material for Energy expenditure and dietary intake in research: A visualization analysis [file sj-pdf-2-nah-10.1177_02601060251404993.pdf]
