# Supplementary material for: Energy expenditure and dietary intake in research: A visualization analysis
Source: Nutr Health. 2026 Jan 19;32(3):777–86. doi: 10.1177/02601060251404993 (PMC13144660; doi:10.1177/02601060251404993)
Supplement: sj-pdf-3-nah-10.1177_02601060251404993 - Supplemental material for Energy expenditure and dietary intake in research: A visualization analysis [file sj-pdf-3-nah-10.1177_02601060251404993.pdf]

Supplemental file 3. Top 100 keywords for a) all publications, b) animal models, and c) human models. Colours in tables are associated with cluster colours in Figure 2.

a) Keywords – All Publications

id	Keywords	x	y	Cluster	Number of Links	Total link strength	Total Number of Occurrences	Avg. pub. year	Avg. citations	Avg. norm. citations
5904	energy expenditure	0.445	-0.3996	1	98	4317	1005	2018.1592	24.5114	0.9438
2364	body composition	0.9983	-0.2874	1	88	2101	537	2018.0987	18.8007	0.6913
19793	weight loss	0.7606	-0.2686	1	97	1989	442	2018.0136	22.9163	0.8313
14735	physical activity	1.1901	0.2355	1	85	1772	449	2018.0713	19.0535	0.7392
6314	exercise	0.6379	-0.1127	1	97	1763	424	2018.4269	18.5165	0.7347
4962	diet	0.5069	0.1497	1	97	1599	390	2018.4462	23.2641	0.9122
15630	protein	0.2581	-0.407	1	97	1366	333	2018.7928	21.982	0.9872
5879	energy balance	0.5061	0.5136	1	97	1197	266	2018.2256	24.6278	0.9183
13964	overweight	1.0185	0.1984	1	88	1187	260	2018.5308	20.1385	0.8682
13445	nutrition	0.8786	-0.158	1	87	1097	331	2018.5861	15.8489	0.6922
16667	risk	0.6562	0.0385	1	95	1029	287	2018.6307	21.4286	0.8882
19784	weight	0.7136	0.4731	1	96	970	217	2018.0323	18.6682	0.7452
9515	indirect calorimetry	1.0906	-0.4958	1	84	958	262	2018.3015	14.6069	0.5815
6369	expenditure	1.0626	-0.1226	1	87	934	240	2017.5208	21.8667	0.7344
5918	energy intake	1.0857	0.3412	1	84	890	206	2018.1019	17.6408	0.6376
8329	health	0.6605	0.185	1	94	877	224	2019.4464	18.3973	1.0178
1626	association	0.5675	0.3424	1	95	807	190	2019.0053	24.5895	1.0741
16524	resting metabolic rate	1.2711	-0.284	1	73	727	171	2018.1228	20.5906	0.713

16519	resting energy expenditure	1.1672	-0.3934	1	80	722	215	2018.186	14.8791	0.5585
3536	children	1.2062	0.098	1	79	716	206	2017.801	15	0.6035
763	adults	1.2144	-0.1275	1	72	705	178	2018.4607	15.5393	0.6115
19966	women	1.0632	0.0138	1	81	639	155	2017.9484	20.9613	0.812
2390	body mass index	0.883	0.0653	1	88	638	166	2018.1506	18.259	0.6694
11800	metabolic rate	0.8992	-0.4432	1	85	631	174	2017.9138	16.0977	0.5927
3011	carbohydrate	0.7157	-0.463	1	80	601	141	2018.0638	20.539	0.7827
19489	validation	1.3413	-0.0793	1	66	595	164	2017.7805	16.6585	0.5903
13991	oxidation	0.3083	-0.579	1	93	589	126	2017.9365	22.5079	0.8537
1476	appetite	0.885	0.4629	1	75	584	127	2018.4724	18.5827	0.8176
8099	growth	0.3167	-0.0073	1	93	547	173	2018.3699	22	0.9241
9033	humans	0.5665	-0.5889	1	90	547	130	2017.3615	29.5923	1.0621
5870	energy	0.3106	-0.2376	1	95	528	139	2018.8993	20.0504	0.9389
15427	prevalence	0.8563	0.2766	1	83	497	121	2018.7769	18.2727	0.7559
14404	performance	0.617	-0.2807	1	79	484	164	2018.5427	15.6951	0.6599
9737	insulin resistance	-0.3413	-0.0391	2	98	5275	1125	2018.2462	28.4436	1.1037
11859	metabolism	-0.1082	-0.1221	2	98	4081	997	2018.5216	27.4293	1.1298
9611	inflammation	-0.5098	0.2862	2	98	2957	628	2019.1465	27.1672	1.2293
8657	high fat diet	-0.5028	0.6178	2	97	2020	412	2018.4782	25.5801	1.0564
17506	skeletal muscle	-0.0774	-0.397	2	98	2011	461	2017.7093	30.269	1.0429

7775	glucose	-0.0158	0.1407	2	98	1913	426	2018.8028	26.8545	1.1134
10898	liver	-0.2625	-0.2144	2	97	1484	318	2018.6761	25.1855	1.0323
11825	metabolic syndrome	0.0235	-0.185	2	97	1474	339	2017.7965	25.3451	0.8905
5924	energy metabolism	0.164	0.0487	2	98	1370	352	2018.5114	19.5625	0.7913
9744	insulin sensitivity	-0.143	-0.6799	2	97	1344	299	2018.3478	28.3378	1.1615
14010	oxidative stress	-0.3404	0.4112	2	91	1306	363	2018.551	23.8072	0.9931
16449	resistance	-0.0855	0.2864	2	97	1251	272	2018.4412	31.1029	1.1171
10794	lipid metabolism	-0.3002	-0.502	2	92	1223	263	2018.4487	29.6882	1.1635
8197	gut microbiota	-0.1492	0.4463	2	93	1072	270	2020.0593	30.4148	1.8023
11554	mechanisms	-0.3428	0.1724	2	96	1026	231	2018.8658	25.658	1.106
5276	disease	0.2124	-0.1199	2	98	916	249	2018.743	26.1124	1.1268
8540	hepatic steatosis	-0.4089	-0.6653	2	88	838	177	2018.8814	27.5085	1.2464
12170	mitochondria	-0.6209	0.0345	2	91	797	185	2018.7784	26.1514	1.0867
16092	rats	-0.2755	0.6195	2	93	756	166	2017.7229	30.8133	1.0568
12610	muscle	0.0872	-0.5914	2	93	665	153	2018.3203	27.085	1.0289
4894	diabetes	-0.1819	0.0483	2	93	630	125	2018.032	26.16	1.0109
9573	induced obesity	-0.3916	0.7585	2	84	622	129	2017.9225	26.7054	1.0113

5516	dysfunction	-0.5066	0.4404	2	87	608	139	2018.7482	24.705	1.1499
18211	supplementation	0.2632	0.2911	2	96	600	146	2018.8288	19.4315	0.8396
2532	brain	-0.1248	0.7594	2	87	565	140	2018.1429	25.5214	1.0156
9661	inhibition	-0.6481	0.4797	2	88	564	130	2018.7231	29.2385	1.2322
18039	stress	-0.0583	0.5622	2	95	558	148	2019.0946	22.6824	1.1291
4706	deficiency	0.1087	0.2614	2	90	513	123	2018.4797	22.0813	0.9616
3584	cholesterol	0.105	-0.3381	2	88	503	117	2018.6239	28.0427	1.1952
11883	metabolomics	0.2896	0.1786	2	77	371	124	2018.9355	20.3629	0.9018
13599	obesity	0.002	-0.0198	3	98	10076	2201	2018.761	23.2326	1.0067
652	adipose tissue	-0.6447	-0.1501	3	98	3771	787	2018.4574	28.9975	1.1678
6390	expression	-0.6565	0.2158	3	96	3486	785	2018.4611	26.9554	1.1036
18623	thermogenesis	-0.7599	-0.3677	3	98	2776	534	2019.0094	24.0618	1.1307
2656	brown adipose tissue	-0.8273	-0.2035	3	96	2615	524	2018.8206	25.4733	1.0909
11973	mice	-0.7619	0.1171	3	97	2528	534	2018.118	34.1124	1.311
6532	fat	-0.0612	-0.5633	3	98	2418	502	2018.4143	31.1494	1.2242
445	activation	-0.9129	0.4538	3	93	1906	409	2018.6528	35.4377	1.3823
7523	gene expression	-0.472	-0.1039	3	96	1781	440	2017.9727	30.4545	1.0686
610	adipocytes	-0.9735	-0.4376	3	87	1564	287	2018.7143	31.6725	1.2455

4984	diet induced obesity	-0.7279	0.3615	3	95	1271	269	2017.974	35.5613	1.2283
16128	receptor	-0.6823	0.6368	3	90	935	211	2018.3175	33.0237	1.4247
2643	brown	-1.0309	-0.286	3	79	926	174	2019.7241	24.7126	1.3242
612	adipogenesis	-1.15	-0.0641	3	88	869	157	2019.1274	24.3248	1.1264
7792	glucose homeostasis	-0.412	-0.2881	3	92	865	176	2018.0682	37.1364	1.353
8864	homeostasis	-0.273	0.2877	3	97	864	186	2018.9301	29.0215	1.2623
7521	gene	-0.5753	-0.274	3	88	805	189	2018.3016	27.4286	1.2305
3368	cells	-0.8978	0.3103	3	88	792	190	2018.7474	26.4579	1.2815
19874	white adipose tissue	-1.0097	-0.0085	3	83	792	145	2019.0552	21.9241	0.9817
5202	differentiation	-1.1692	0.1185	3	84	745	152	2018.5789	27.2171	1.1224
12490	mouse	-0.9845	0.1756	3	89	722	145	2018.4621	31.1241	1.2351
19282	ucp1	-1.1522	-0.4171	3	76	713	122	2018.9262	26.377	1.0751
9263	identification	-0.4854	0.101	3	93	698	172	2018.4186	38.4826	1.4717
15271	ppar-gamma	-1.1425	-0.2135	3	78	685	140	2018.2571	33.2786	1.2165
10843	lipolysis	-0.6276	-0.7473	3	82	664	133	2019.2632	21.2932	1.0724
1131	ampk	-0.633	-0.4486	3	84	660	142	2019.7183	24	1.1812
19866	white	-0.9029	-0.5808	3	85	656	119	2019.395	30.3866	1.3674

18754	tissue	-0.4836	-0.5345	3	90	650	141	2018.9149	26.7447	1.1519
434	activated protein-kinase	-0.6813	-0.5937	3	83	631	137	2017.4891	36.219	1.2101
9446	in-vivo	-0.8054	-0.0481	3	80	481	128	2016.9219	35.9531	1.1135
7069	food intake	0.3593	0.6929	4	94	1935	431	2017.9026	23.7796	0.9203
2409	body weight	0.2871	0.5463	4	97	1661	355	2017.9127	27.6028	1.1139
10607	leptin	0.0936	0.6856	4	97	1294	270	2018.2556	18.2852	0.7918
9726	insulin	0.0702	0.4337	4	98	1193	257	2018.2412	21.4047	0.9163
624	adiponectin	-0.2328	-0.363	4	92	658	120	2018.65	28.975	1.0564
9220	hypothalamus	-0.1996	0.9286	4	76	588	116	2018.5862	18.3362	0.795

b) Keywords – Animal Publications

id	Keywords	x	y	Cluster	Number of Links	Total link strength	Total Number of Occurrences	Avg. pub. year	Avg. citations	Avg. norm. citations
3745	expression	0.1919	0.087	1	101	3370	674	2018.439	27.2166	0.9757
7042	metabolism	-0.2025	0.2571	1	101	3202	673	2018.63	29.7311	1.0759
5703	inflammation	0.051	0.4671	1	101	2730	518	2019.183	29.3012	1.1321
7114	mice	-0.013	-0.061	1	101	2510	500	2018.174	33.286	1.1124
6499	liver	-0.4635	0.5399	1	100	1387	267	2018.644	27.4157	0.974
8313	oxidative stress	-0.2898	0.9376	1	97	1106	262	2018.428	24.916	0.8858
4839	gut microbiota	-0.8185	0.3978	1	97	943	215	2020.056	31.0186	1.6037

6861	mechanisms	-0.1207	0.7541	1	101	894	174	2018.874	29.7011	1.09
3499	energy metabolism	-0.0442	0.2861	1	100	874	187	2018.38	22.5187	0.7967
3019	diet	-0.8929	0.0793	1	98	767	168	2018.536	24.3095	0.9238
7226	mitochondria	0.3088	0.7059	1	97	754	153	2018.712	27.3399	0.9705
5239	homeostasis	0.1333	-0.0831	1	101	753	146	2018.98	28	1.1237
2068	cells	0.5294	0.6877	1	96	732	157	2018.713	28.0255	1.1461
3155	disease	-0.4595	0.8615	1	93	660	158	2018.949	25.0443	0.9656
3292	dysfunction	-0.0125	0.6425	1	95	586	124	2018.839	25.0645	1.041
5731	inhibition	-0.2633	0.5744	1	98	563	117	2018.709	29.735	1.0918
10575	stress	-0.1326	0.0938	1	96	470	102	2019.353	21.3039	1.0279
10675	supplementation	-0.601	0.365	1	94	412	86	2018.605	21.1047	0.8077
2854	deficiency	-0.1304	0.4881	1	89	401	76	2018.487	21.0526	0.8138
8467	pathway	-0.2938	0.7588	1	83	387	79	2018.481	26.2152	1.0288
9838	risk	-0.7519	0.2561	1	90	366	95	2018.716	23.1895	0.8739
2193	cholesterol	-0.5787	0.7282	1	80	364	80	2018.65	23.95	1.007
1113	autophagy	0.2047	0.885	1	84	357	73	2019.11	41.5479	1.4578
4905	health	-0.6931	0.5996	1	83	353	84	2019.845	24.5476	1.1666
1035	association	-0.4292	0.3409	1	85	350	73	2019.014	28.7945	1.2597
241	acid	-0.852	0.5635	1	81	347	78	2019.192	20.1538	0.7919

8301	oxidation	-0.6278	0.1499	1	86	346	68	2018.441	21.2206	0.7815
4770	growth	-0.3155	0.1029	1	86	307	68	2018.324	30.7059	1.0865
7133	microbiota	-0.9563	0.3443	1	82	295	66	2020.197	28.0455	1.3691
7058	metabolomics	-0.7545	0.813	1	68	231	71	2018.803	22.1972	0.829
3489	energy expenditure	0.1605	-0.7596	2	101	2478	496	2018.218	30.6028	1.0273
5099	high fat diet	-0.3937	-0.2518	2	100	2132	406	2018.434	24.7069	0.8996
4387	gene expression	0.2961	-0.4301	2	100	1558	328	2017.902	35.122	1.0595
4555	glucose	-0.4619	0.1213	2	101	1443	283	2018.774	27.1166	1.016
4138	food intake	-0.7894	-0.7436	2	96	1417	294	2017.946	23.0136	0.8097
3029	diet induced obesity	0.0363	-0.4898	2	101	1367	265	2018.008	32.2868	1.0112
1486	body weight	-0.3354	-0.8815	2	96	1153	230	2018	26.9043	1.0323
6311	leptin	-0.4841	-0.7923	2	99	1072	200	2018.38	18.51	0.7456
9700	resistance	-0.287	-0.3853	2	100	1058	201	2018.403	32.4527	0.9983
7023	metabolic syndrome	-0.5622	-0.0581	2	100	1057	223	2017.915	25.8251	0.8153
5773	insulin	-0.6251	-0.2383	2	101	934	183	2018.366	20.8251	0.8339
9516	receptor	-0.1574	-0.2626	2	101	918	185	2018.335	34.0919	1.2802
9221	protein	-0.1134	-0.4491	2	98	877	179	2018.899	26.1732	1.0162

4565	glucose homeostasis	-0.0778	-0.7969	2	97	852	163	2018.092	36.5276	1.1845
9498	rats	-0.7343	-0.0203	2	99	680	141	2017.645	28.5177	0.8955
3481	energy balance	-0.6077	-0.717	2	92	654	134	2018.134	27.1119	0.8541
5683	induced obesity	-0.2813	-0.6417	2	93	613	123	2017.943	26.5041	0.8789
5468	hypothalamus	-0.7071	-0.9676	2	79	580	104	2018.519	19.1154	0.7028
11619	weight loss	-0.4381	-0.5849	2	91	573	123	2018.252	22.2276	0.7612
1552	brain	-0.6909	-0.4785	2	91	539	120	2018.333	23.7333	0.8644
405	adiponectin	-0.4972	-0.3768	2	94	535	90	2018.7	33.4778	1.0612
7790	neurons	-0.5174	-0.9844	2	76	445	96	2018.427	28.6458	0.9852
11614	weight	-1.034	-0.1856	2	85	412	89	2018.191	21.9438	0.8278
3495	energy homeostasis	-0.187	-1.0098	2	78	402	78	2017.885	27.2564	0.7821
9472	rat	-0.8349	-0.3129	2	82	348	78	2017.256	24.8333	0.6967
6952	messenger-rna	0.0217	-1.0327	2	74	332	72	2017.042	37.9167	1.0684
1468	body composition	-0.9805	-0.4442	2	68	331	80	2017.213	18.7375	0.512
10028	secretion	-0.8567	-0.148	2	86	320	68	2017.515	28.5441	0.8834
8096	obesity	0.0263	-0.2371	3	101	7899	1561	2018.901	24.3876	0.9465
433	adipose tissue	0.3989	-0.0244	3	101	3282	630	2018.605	27.9175	1.0167

10923	thermogenesis	0.7075	-0.4168	3	101	2610	455	2019.244	24.7341	1.0393
1622	brown adipose tissue	0.419	-0.8069	3	101	2450	445	2018.921	25.5393	0.9499
292	activation	0.4088	-0.1793	3	99	1877	362	2018.674	33.953	1.1843
3830	fat	0.7199	-0.1171	3	100	1815	337	2018.644	33.6231	1.2097
393	adipocytes	0.9434	-0.1166	3	98	1611	263	2018.715	31.7681	1.0804
1609	brown	0.9302	-0.2726	3	94	963	164	2019.774	25.2744	1.157
395	adipogenesis	0.83	0.2861	3	97	874	145	2019.172	21.5655	0.9196
11659	white adipose tissue	0.5269	-0.6046	3	96	801	132	2019.023	21.5758	0.8372
7425	mouse	0.5165	-0.4167	3	96	739	137	2018.489	31.3212	1.0885
3116	differentiation	0.9405	0.4404	3	93	726	136	2018.61	26.1838	0.9489
11349	ucp1	0.9854	-0.4679	3	89	714	111	2018.928	25.3423	0.9063
4385	gene	0.5786	0.0196	3	94	701	146	2018.343	26.5479	1.01
9032	ppar-gamma	0.9172	0.1083	3	90	682	127	2018.244	33.0945	1.0534
6467	lipolysis	0.713	0.1386	3	92	668	121	2019.504	21.7025	0.9522
11651	white	0.8863	-0.5933	3	90	664	108	2019.407	30.5556	1.181
1653	browning	1.1809	-0.2691	3	85	628	95	2019.863	16.3684	0.7688
11002	tissue	0.5624	-0.2358	3	98	624	123	2019.033	27.4634	1.0387
1205	beige	1.188	-0.0797	3	74	425	70	2019.443	31.9143	1.3183
1213	beige fat	0.7433	-0.8389	3	77	410	69	2018.87	38.9565	1.4021

347	adaptive thermogenesis	0.7176	-0.6654	3	78	366	66	2017.818	39.2121	1.2216
3478	energy	0.0221	-0.6263	3	92	356	69	2018.855	23.087	0.9007
622	alpha	0.3922	0.1486	3	84	354	66	2018.53	22.2879	0.8953
5783	insulin resistance	0.1762	0.2893	4	101	4759	923	2018.293	29.3402	1.0025
10289	skeletal muscle	0.2453	0.4833	4	100	1452	296	2017.608	32.625	0.9859
6428	lipid metabolism	-0.2736	0.3915	4	99	1176	231	2018.377	31.0216	1.0547
5788	insulin sensitivity	0.2697	-0.6227	4	94	1076	216	2018.463	29.4954	1.0759
5018	hepatic steatosis	0.0276	0.8652	4	97	817	159	2018.868	26.7547	1.0559
729	ampk	0.4506	0.8586	4	95	691	130	2019.654	24.5	1.0532
281	activated protein-kinase	0.6286	0.4119	4	94	642	122	2017.623	35.5492	1.052
5491	identification	0.5176	0.2516	4	92	597	123	2018.423	39.5285	1.3292
3718	exercise	0.0502	0.1622	4	95	593	118	2018.246	22.5678	0.7803
2975	diabetes	-0.2292	-0.0964	4	92	571	104	2018.058	26.1346	0.8864
8639	pgc-1-alpha	1.0108	0.2749	4	83	511	81	2018.333	29.2222	1.0681
9028	ppar-alpha	0.2435	-0.2094	4	87	494	95	2017.463	47.4316	1.3964
10090	sensitivity	-0.3944	-0.0707	4	91	489	91	2018.022	29.6264	0.9274
4014	fgf21	0.145	-0.3776	4	87	481	88	2018.682	28.3182	1.0593
5608	in-vivo	0.3861	0.3687	4	90	473	114	2017.079	32.2807	0.9399
7501	muscle	0.1572	0.6688	4	91	468	93	2018.215	32.6022	1.0803
7242	mitochondrial biogenesis	0.7025	0.5474	4	86	466	83	2018.06	30.7952	0.9977
8748	phosphorylation	0.6661	0.7897	4	83	432	83	2018.602	27.4819	0.9397
3914	fatty liver	-0.09	1.0114	4	81	354	73	2018.137	24.9315	0.8392

3921	fatty-acid oxidation	0.4731	0.5279	4	77	304	66	2017.742	34.2121	1.1205

c) Keywords – Human Publications

id	Keywords	x	y	Cluster	Number of Links	Total link strength	Total Number of Occurrences	Avg. pub. year	Avg. citations	Avg. norm. citations
6696	metabolism	-0.4734	-0.0256	1	98	1162	324	2018.296	22.6543	1.1178
5513	insulin resistance	-0.6307	-0.3791	1	98	866	202	2018.035	24.3515	1.0853
8837	protein	-0.1938	0.0396	1	95	659	154	2018.669	17.1104	0.9715
4473	glucose	-0.7239	0.0172	1	92	604	143	2018.86	26.3357	1.2478
3364	energy metabolism	-0.2743	-0.0906	1	98	599	156	2018.667	16.0385	0.7859
9904	skeletal muscle	-0.7489	0.1506	1	87	531	135	2017.941	26.1407	1.1259
303	adipose tissue	-0.9322	0.1241	1	84	490	120	2018.033	30.55	1.3856
8124	performance	-0.2702	0.1661	1	87	488	144	2018.576	15.9444	0.8227
5440	inflammation	-0.5624	-0.6477	1	90	427	110	2018.973	17.1182	1.0706
10531	thermogenesis	-0.8719	0.5101	1	81	362	79	2017.658	20.1899	0.8514
2946	disease	-0.2019	-0.5562	1	87	350	91	2018.385	27.989	1.414
5518	insulin sensitivity	-0.753	0.3246	1	82	348	83	2018.048	25.3494	1.2139
5509	insulin	-0.6189	0.2367	1	84	330	74	2017.932	22.8378	0.9931

3658	expression	-0.9063	-0.0872	1	79	306	111	2018.595	25.3694	1.1837
4352	gene expression	-1.0913	-0.0952	1	67	300	112	2018.179	16.8125	0.7886
5964	leptin	-0.515	0.3646	1	80	300	70	2017.9	17.6571	0.7147
9282	resistance	-0.5072	0.0999	1	85	288	71	2018.549	27.2958	1.262
4646	growth	-0.3752	-0.3819	1	79	282	105	2018.4	16.3714	0.8901
7889	oxidation	-0.5426	0.626	1	71	277	58	2017.345	24.069	0.97
7094	muscle	-0.4422	-0.1731	1	82	261	60	2018.483	18.55	0.8356
7901	oxidative stress	-0.8081	-0.42	1	72	253	101	2018.871	20.9406	1.1814
1548	caloric restriction	-0.374	0.3108	1	73	248	52	2018.596	30.1346	1.3876
9319	responses	-0.6834	0.5061	1	69	224	57	2018.579	18.7719	1.1041
1582	cancer	-0.0371	-0.576	1	73	220	54	2019.093	26.2037	1.3692
6065	lipid metabolism	-1.0083	-0.2007	1	67	218	56	2018.375	27.3929	1.2437
10312	supplementation	-0.5213	-0.3019	1	75	216	60	2019.15	17.0333	0.9016
8401	plasma	-0.6247	-0.1465	1	77	191	50	2017.94	17.56	0.8564
1447	brown adipose tissue	-1.0697	0.4198	1	52	181	56	2018.054	24.6786	1.2805
6504	mechanisms	-0.785	-0.1977	1	67	175	57	2018.842	13.3158	0.8258
6122	liver	-0.956	-0.3919	1	60	173	51	2018.843	13.5294	0.6987

4690	gut microbiota	-0.8889	-0.5982	1	55	162	55	2020.073	28.1091	1.9711
6710	metabolomics	-0.7201	-0.6014	1	57	150	53	2019.113	17.9245	1.0185
2592	deficiency	-0.1989	-0.3315	1	57	145	47	2018.468	23.766	1.1519
197	activation	-1.0734	0.0603	1	55	139	47	2018.489	46.8723	1.7698
10213	stress	-0.5312	-0.4918	1	60	138	46	2018.522	25.7391	1.2513
5249	identification	-0.8754	-0.2894	1	57	129	49	2018.408	35.8571	1.5593
1257	body composition	0.2481	0.2144	2	99	2191	457	2018.254	18.8228	0.8886
5388	indirect calorimetry	0.8892	0.062	2	92	1075	224	2018.42	14.4062	0.7156
3645	expenditure	0.6207	0.5876	2	93	1024	206	2017.612	21.4854	0.9176
9325	resting energy expenditure	0.7167	-0.0166	2	92	907	199	2018.106	15.2915	0.707
361	adults	0.7919	-0.2917	2	90	847	163	2018.288	16.0859	0.7521
9330	resting metabolic rate	0.5152	0.7655	2	89	836	161	2018.162	20.6087	0.9063
11000	validation	1.0132	0.2718	2	91	792	157	2017.803	16.9427	0.7655
11313	women	0.4263	0.5055	2	91	693	140	2017.864	20.5357	0.9125
6657	metabolic rate	0.5807	0.2119	2	91	605	140	2018.064	14.4	0.6683
11003	validity	0.9763	0.5274	2	74	538	104	2018.337	17.8365	0.8435
3779	fat-free mass	0.366	0.8857	2	80	459	84	2018.536	16.1786	0.8277
5134	humans	0.0154	0.6028	2	88	422	87	2017.483	25.2644	1.1525
3025	doubly labeled water	0.8117	0.7211	2	71	414	76	2017.29	23.1711	0.9599
7634	obese	0.2484	0.4202	2	86	412	79	2017.924	21.2405	0.9075

6565	men	-0.0772	0.3194	2	86	407	80	2017.313	21.25	0.9993
9271	requirements	1.0963	0.0324	2	72	396	76	2018.263	15.6184	0.9815
974	basal metabolic rate	0.8265	0.343	2	77	383	82	2018.11	19.1951	1.113
6403	mass	0.3737	-0.1632	2	82	347	71	2018.817	14.4366	0.8442
8667	predictive equations	1.271	-0.0714	2	57	311	56	2018.536	9.6071	0.4597
149	accuracy	1.2712	0.3119	2	54	265	48	2018.333	13.7708	0.6369
8517	population	1.0438	-0.2474	2	69	258	49	2017.245	15.5918	0.6517
2867	dietary-intake	0.7499	0.1697	2	68	244	47	2018.426	19.6596	0.8832
401	age	0.0661	-0.1921	2	74	228	51	2018.118	16.9216	0.9468
7651	obesity	-0.0432	-0.0316	3	100	2968	640	2018.419	20.425	0.9967
8268	physical activity	0.4253	0.1472	3	94	1857	392	2018.046	18.7602	0.894
7869	overweight	0.498	-0.0272	3	97	1132	206	2018.422	17.3641	0.8564
2726	diet	-0.1264	-0.1756	3	96	967	222	2018.378	22.482	0.9858
3359	energy intake	0.6649	0.4171	3	91	963	181	2017.983	18.2044	0.8034
1916	children	0.6377	-0.1683	3	92	862	187	2017.85	14.9893	0.7765
9401	risk	0.0263	-0.7278	3	94	833	192	2018.589	20.5677	1.0363
4768	health	0.1517	-0.6399	3	94	650	140	2019.207	14.7143	1.0564
11187	weight	0.1858	-0.0708	3	93	650	128	2017.922	16.4062	0.7516
1280	body mass index	0.5891	-0.3812	3	92	617	130	2017.954	17.3615	0.7438

835	association	0.147	-0.8534	3	89	525	117	2019	21.9744	1.0697
6676	metabolic syndrome	-0.4294	-0.7836	3	79	489	116	2017.569	24.4224	1.0026
332	adolescents	0.9122	-0.1166	3	79	473	89	2017.472	16.2809	0.6546
8715	prevalence	0.4842	-0.6887	3	75	422	87	2018.678	18.8046	0.9153
4053	food	0.2903	0.5988	3	79	327	72	2017.944	17.8194	0.819
2317	consumption	0.0037	-0.4135	3	77	312	67	2018.194	20.6716	0.8972
9411	risk-factors	0.1393	-0.5005	3	78	260	58	2018.431	17.931	0.8607
6603	metaanalysis	-0.1979	-0.7499	3	74	236	52	2019.154	13.5385	0.924
1715	cardiovascular-disease	-0.0784	-0.884	3	63	214	46	2018.413	17.5652	0.9079
3348	energy expenditure	0.101	0.4126	4	99	2350	509	2018.102	18.5776	0.9006
11195	weight loss	-0.1247	0.4595	4	95	1625	319	2017.922	23.1912	1.0083
3601	exercise	-0.0148	0.171	4	98	1387	306	2018.497	16.9673	0.8562
3737	fat	-0.3534	0.5009	4	93	790	165	2017.946	26.097	1.0895
3327	energy balance	0.1367	0.6946	4	95	659	132	2018.318	22.1136	1.0646
4079	food intake	0.0413	0.866	4	89	648	137	2017.81	25.438	1.1282
1294	body weight	-0.2589	0.5978	4	90	616	125	2017.752	28.904	1.1885
744	appetite	-0.1997	0.9428	4	79	528	98	2018.541	19.3061	1.0713
1623	carbohydrate	-0.3693	0.7285	4	84	486	99	2017.929	22.3434	0.9573

10259	substrate oxidation	-0.601	0.8052	4	69	263	51	2018.196	17.1373	0.7446
11219	weight-gain	-0.1023	0.7264	4	69	259	52	2017.096	28.4231	1.2821
3764	fat oxidation	-0.4703	0.9616	4	60	242	46	2017.174	19.3043	0.791
7543	nutrition	0.3997	-0.3172	5	97	1118	285	2018.561	16.4	0.865
7613	nutritional status	0.621	-0.5605	5	72	398	98	2018.857	10.1327	0.5183
947	balance	0.4551	0.3574	5	84	391	77	2017.649	31.5325	1.2482
6357	malnutrition	0.7411	-0.674	5	71	359	84	2019.024	9.631	0.5844
7009	mortality	0.5874	-0.7881	5	74	351	85	2018.671	15.1059	0.9216
5304	impact	0.2337	-0.3688	5	82	332	75	2018.733	18.1467	1.1091
4672	guidelines	0.8371	-0.4615	5	66	259	58	2018.224	17.7414	0.8735
3319	energy	0.2779	0.0476	5	74	251	70	2018.943	17.0857	1.0886
6369	management	0.3476	-0.5629	5	73	212	48	2019	16.5625	0.9258
9538	sarcopenia	0.4322	-0.9013	5	59	205	48	2019.542	12.5833	0.7483
3405	enteral nutrition	0.9516	-0.5309	5	48	176	50	2017.44	16.18	0.7621
